# 4-(4-Meth­oxy­benzene­sulfonamido)­benzoic acid

**DOI:** 10.1107/S1600536811025098

**Published:** 2011-06-30

**Authors:** Islam Ullah Khan, Ghulam Mustafa, Mehmet Akkurt

**Affiliations:** a Materials Chemistry Laboratory, Department of Chemistry, GC University, Lahore 54000, Pakistan; b Department of Physics, Faculty of Sciences, Erciyes University, 38039 Kayseri, Turkey

## Abstract

The asymmetric unit of the title compound, C_14_H_13_NO_5_S, contains two independent mol­ecules in which the dihedral angles between the aromatic rings are 83.45 (11) and 86.65 (9)°. In the crystal, the independent mol­ecules are connected by N—H⋯O and O—H⋯O hydrogen bonds, forming a double-chain structure along [401]. A weak π–π stacking inter­action with a centroid–centroid distance of 3.7509 (13) Å and C—H⋯O hydrogen bonds are also observed.

## Related literature

For background to the biological activity of sulfonamides, see: Hanson *et al.* (1999 ▶). For related structures, see: Mustafa *et al.* (2010 ▶, 2011 ▶). For bond-length data, see: Allen *et al.* (1987 ▶).
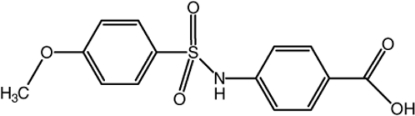


## Experimental

### 

#### Crystal data

C_14_H_13_NO_5_S
*M* *_r_* = 307.32
Monoclinic, 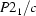

*a* = 8.6980 (3) Å
*b* = 21.7471 (8) Å
*c* = 14.5824 (6) Å
β = 95.153 (2)°
*V* = 2747.21 (18) Å^3^
*Z* = 8
Mo *K*α radiation
μ = 0.26 mm^−1^
*T* = 296 K
0.38 × 0.31 × 0.28 mm

#### Data collection

Bruker APEXII CCD diffractometer
Absorption correction: multi-scan (*SADABS*; Bruker 2007 ▶) *T*_min_ = 0.909, *T*_max_ = 0.931
25188 measured reflections
6806 independent reflections
5475 reflections with *I* > 2σ(*I*)
*R*_int_ = 0.018

#### Refinement

*R*[*F* ^2^ > 2σ(*F* ^2^)] = 0.044
*wR*(*F* ^2^) = 0.120
*S* = 1.04
6806 reflections
394 parameters
4 restraints
H atoms treated by a mixture of independent and constrained refinement
Δρ_max_ = 0.45 e Å^−3^
Δρ_min_ = −0.30 e Å^−3^

### 

Data collection: *APEX2* (Bruker, 2007 ▶); cell refinement: *SAINT* (Bruker, 2007 ▶); data reduction: *SAINT*; program(s) used to solve structure: *SIR97* (Altomare *et al.*, 1999 ▶); program(s) used to refine structure: *SHELXL97* (Sheldrick, 2008 ▶); molecular graphics: *ORTEP-3 for Windows* (Farrugia, 1997 ▶); software used to prepare material for publication: *WinGX* (Farrugia, 1999 ▶) and *PLATON* (Spek, 2009 ▶).

## Supplementary Material

Crystal structure: contains datablock(s) global, I. DOI: 10.1107/S1600536811025098/is2741sup1.cif

Structure factors: contains datablock(s) I. DOI: 10.1107/S1600536811025098/is2741Isup2.hkl

Supplementary material file. DOI: 10.1107/S1600536811025098/is2741Isup3.cml

Additional supplementary materials:  crystallographic information; 3D view; checkCIF report

## Figures and Tables

**Table 1:** Hydrogen-bond geometry (Å, °)

*D*—H⋯*A*	*D*—H	H⋯*A*	*D*⋯*A*	*D*—H⋯*A*
N1—H*N*1⋯O8^i^	0.84 (2)	2.31 (2)	3.122 (2)	165 (2)
O2—H*O*1⋯O7^ii^	0.81 (2)	1.78 (2)	2.583 (2)	172 (2)
N2—H*N*2⋯O3^iii^	0.82 (2)	2.15 (2)	2.959 (2)	173 (2)
O6—H*O*2⋯O1^iv^	0.82 (2)	2.02 (2)	2.8338 (19)	173 (2)
C4—H4⋯O4	0.93	2.54	3.149 (2)	123
C9—H9⋯O7	0.93	2.32	3.224 (3)	165
C13—H13⋯O10^v^	0.93	2.57	3.381 (3)	146
C14—H14*A*⋯O8^iv^	0.96	2.42	3.370 (3)	169
C23—H23⋯O2	0.93	2.52	3.286 (2)	140
C26—H26⋯O1^vi^	0.93	2.57	3.197 (2)	125

Symmetry codes: (i) 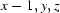; (ii) 
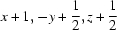
; (iii) 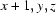; (iv) 
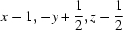
; (v) 
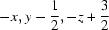
; (vi) 
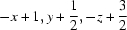
.

**Fig. 1.**
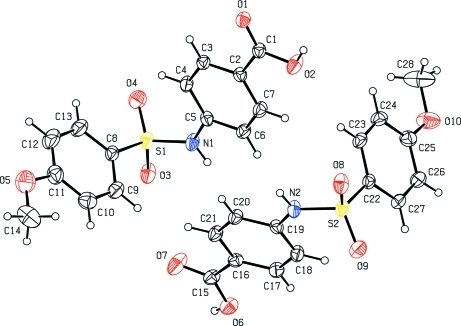
The asymmetric unit of the title compound, showing the labelling of all non-H atoms. Displacement ellipsoids for non-H atoms are drawn at the 30% probability level.

**Fig. 2.**
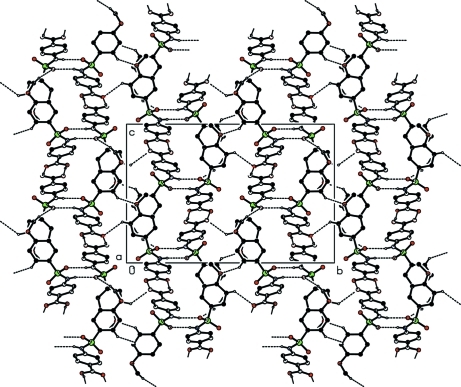
A packing diagram of the title compound with hydrogen bonds, viewed down a axis. Hydrogen atoms that not involved in the hydrogen-bonding (dashed lines) have been omitted for clarity.

## supplementary crystallographic information

### Comment

Sulfonamides are well known for their various types of biological activities (*e.g.* Hanson *et al.*, 1999). In the present paper, the structure of the title compound, (I), is reported.

As shown in Fig. 1, the asymmetric unit of the title compound (I) contains two crystallographically independent molecules A (with S1) and B (with S2). All the bond lengths (Allen *et al.*, 1987) and angles are within normal ranges and they are similar to those of the related molecules (Mustafa *et al.*, 2010, 2011). The dihedral angles between the aromatic rings of (I) is 83.45 (11)° for molecule A and 86.65 (9)° for molecule B.

The crystal packing (Fig. 2, Table 1) is stabilized by C—H···O, N—H···O and O—H···O hydrogen bonds and a weak π–π interaction between the benzene rings attached with the methoxy group of the symmetry independent molecules [*Cg*2···*Cg*4^iv^ = 3.7509 (13) Å; symmetry code: (iv) *x* - 1, -*y* + 1/2, *z* - 1/2; *Cg*2 and *Cg*4 are the centroid of the C8–C13 and C22–C27 benzene rings, respectively].

### Experimental

To a mixture of *p*-amino benzoic acid (1.0 g, 7.3 mmoles) and distilled water (10 ml) in a round bottomflask (25 ml) 1*M* aqueous sodium carbonate solution was added to maintain the pH between 8–9. 4-Methoxy benzenesulfonyl chloride (1.51 g, 7.3 mmol) was added to this solution and was kept stirred at room temperature for 5 h. pH of the reaction mixture was adjusted to 1–2, using 1 N HCl and the precipitates obtained were filtered, washed with distilled water, dried and recrystallized from methanol to yield light brown crystals of (I).

### Refinement

Five reflections giving bad agreements with Fc, *viz*. (110), (100), (020), (021) and (011), were omitted during the final cycles of refinement. The H atoms of the NH and OH groups of the two molecules in the asymmetric unit were located in a difference map and refined with the distance restraints N—H = 0.86 (2) Å and O—H = 0.82 (2) Å. Their isotropic displacement parameters were set to be 1.2*U*_eq_(N) for NH groups and 1.5*U*_eq_(O) for OH groups. The aromatic and methyl H atoms were placed in calculated positions [C—H = 0.93 and 0.96 Å], with *U*_iso_ constrained to be 1.5 times *U*_eq_ of the carrier atom for the methyl-H and 1.2 times *U*_eq_ for the remaining H atoms.

### Crystal data

**Table:**

C_14_H_13_NO_5_S	*F*(000) = 1280
*M**_r_* = 307.32	*D*_x_ = 1.486 Mg m^−^^3^
Monoclinic, *P*2_1_/*c*	Mo *K*α radiation, λ = 0.71073 Å
Hall symbol: -P 2ybc	Cell parameters from 9988 reflections
*a* = 8.6980 (3) Å	θ = 2.4–28.4°
*b* = 21.7471 (8) Å	µ = 0.26 mm^−^^1^
*c* = 14.5824 (6) Å	*T* = 296 K
β = 95.153 (2)°	Block, light brown
*V* = 2747.21 (18) Å^3^	0.38 × 0.31 × 0.28 mm
*Z* = 8	

### Data collection

**Table:**

Bruker APEXII CCD diffractometer	6806 independent reflections
Radiation source: sealed tube	5475 reflections with *I* > 2σ(*I*)
graphite	*R*_int_ = 0.018
φ and ω scans	θ_max_ = 28.4°, θ_min_ = 2.8°
Absorption correction: multi-scan (*SADABS*; Bruker 2007)	*h* = −11→11
*T*_min_ = 0.909, *T*_max_ = 0.931	*k* = −24→28
25188 measured reflections	*l* = −14→19

### Refinement

**Table:**

Refinement on *F*^2^	Primary atom site location: structure-invariant direct methods
Least-squares matrix: full	Secondary atom site location: difference Fourier map
*R*[*F*^2^ > 2σ(*F*^2^)] = 0.044	Hydrogen site location: inferred from neighbouring sites
*wR*(*F*^2^) = 0.120	H atoms treated by a mixture of independent and constrained refinement
*S* = 1.04	*w* = 1/[σ^2^(*F*_o_^2^) + (0.0499*P*)^2^ + 1.497*P*] where *P* = (*F*_o_^2^ + 2*F*_c_^2^)/3
6806 reflections	(Δ/σ)_max_ = 0.001
394 parameters	Δρ_max_ = 0.45 e Å^−^^3^
4 restraints	Δρ_min_ = −0.30 e Å^−^^3^

### Special details

**Table:**

Geometry. Bond distances, angles *etc*. have been calculated using the rounded fractional coordinates. All su's are estimated from the variances of the (full) variance-covariance matrix. The cell e.s.d.'s are taken into account in the estimation of distances, angles and torsion angles
Refinement. Refinement on *F*^2^ for ALL reflections except those flagged by the user for potential systematic errors. Weighted *R*-factors *wR* and all goodnesses of fit *S* are based on *F*^2^, conventional *R*-factors *R* are based on *F*, with *F* set to zero for negative *F*^2^. The observed criterion of *F*^2^ > σ(*F*^2^) is used only for calculating -*R*-factor-obs *etc*. and is not relevant to the choice of reflections for refinement. *R*-factors based on *F*^2^ are statistically about twice as large as those based on *F*, and *R*-factors based on ALL data will be even larger.

### Fractional atomic coordinates and isotropic or equivalent isotropic displacement parameters (Å^2^)

**Table:**

	*x*	*y*	*z*	*U*_iso_*/*U*_eq_	
S1	−0.24733 (5)	0.16859 (2)	0.57376 (4)	0.0419 (1)	
O1	0.53350 (14)	0.11383 (6)	0.80744 (9)	0.0418 (4)	
O2	0.57070 (16)	0.21434 (6)	0.79480 (13)	0.0606 (5)	
O3	−0.36648 (15)	0.20947 (6)	0.53547 (12)	0.0562 (5)	
O4	−0.28249 (17)	0.12512 (7)	0.64187 (11)	0.0564 (5)	
O5	−0.0035 (3)	0.02617 (9)	0.27559 (14)	0.0870 (8)	
N1	−0.11308 (18)	0.21531 (7)	0.61562 (13)	0.0469 (5)	
C1	0.4873 (2)	0.16478 (8)	0.78203 (12)	0.0373 (5)	
C2	0.33184 (19)	0.17603 (8)	0.73538 (12)	0.0349 (5)	
C3	0.2252 (2)	0.12892 (8)	0.72183 (12)	0.0371 (5)	
C4	0.0773 (2)	0.14010 (8)	0.68239 (13)	0.0407 (5)	
C5	0.03454 (19)	0.19958 (8)	0.65561 (12)	0.0361 (5)	
C6	0.1406 (2)	0.24698 (8)	0.66846 (13)	0.0417 (5)	
C7	0.2870 (2)	0.23542 (9)	0.70806 (14)	0.0444 (6)	
C8	−0.1707 (2)	0.12793 (8)	0.48507 (13)	0.0396 (5)	
C9	−0.1128 (2)	0.15868 (9)	0.41275 (16)	0.0511 (7)	
C10	−0.0545 (3)	0.12605 (11)	0.34123 (16)	0.0567 (7)	
C11	−0.0575 (3)	0.06309 (11)	0.34217 (17)	0.0599 (8)	
C12	−0.1172 (4)	0.03327 (11)	0.4127 (2)	0.0762 (10)	
C13	−0.1732 (3)	0.06474 (10)	0.48410 (17)	0.0612 (8)	
C14	0.0691 (3)	0.05350 (15)	0.20391 (19)	0.0827 (10)	
S2	0.65408 (5)	0.38949 (2)	0.58411 (3)	0.0410 (1)	
O6	−0.15326 (16)	0.40548 (7)	0.37226 (11)	0.0520 (5)	
O7	−0.15559 (17)	0.30391 (7)	0.37416 (16)	0.0794 (7)	
O8	0.79224 (15)	0.35378 (7)	0.60408 (11)	0.0537 (5)	
O9	0.65854 (17)	0.44420 (7)	0.53070 (11)	0.0550 (5)	
O10	0.3728 (2)	0.45077 (9)	0.92259 (12)	0.0764 (7)	
N2	0.53708 (18)	0.34000 (7)	0.53111 (12)	0.0440 (5)	
C15	−0.0880 (2)	0.35269 (9)	0.38936 (14)	0.0433 (5)	
C16	0.07422 (19)	0.35329 (8)	0.42858 (12)	0.0369 (5)	
C17	0.1530 (2)	0.40609 (8)	0.45785 (13)	0.0419 (5)	
C18	0.3058 (2)	0.40391 (8)	0.49278 (14)	0.0435 (6)	
C19	0.38196 (19)	0.34749 (8)	0.49887 (11)	0.0347 (5)	
C20	0.3038 (2)	0.29444 (8)	0.46977 (13)	0.0395 (5)	
C21	0.1526 (2)	0.29756 (8)	0.43499 (14)	0.0429 (5)	
C22	0.57550 (19)	0.40798 (8)	0.68678 (13)	0.0375 (5)	
C23	0.5632 (2)	0.36265 (8)	0.75171 (14)	0.0430 (5)	
C24	0.4966 (2)	0.37489 (9)	0.83181 (14)	0.0468 (6)	
C25	0.4431 (2)	0.43392 (10)	0.84718 (14)	0.0496 (6)	
C26	0.4594 (3)	0.48007 (9)	0.78309 (15)	0.0513 (7)	
C27	0.5238 (2)	0.46746 (8)	0.70259 (14)	0.0444 (6)	
C28	0.3489 (4)	0.40625 (16)	0.9901 (2)	0.1000 (14)	
HN1	−0.129 (3)	0.2526 (7)	0.6033 (15)	0.0500*	
HO1	0.654 (2)	0.2050 (11)	0.8197 (16)	0.0630*	
H3	0.25340	0.08910	0.73950	0.0450*	
H4	0.00680	0.10800	0.67380	0.0490*	
H6	0.11270	0.28670	0.65020	0.0500*	
H7	0.35730	0.26760	0.71680	0.0530*	
H9	−0.11280	0.20140	0.41180	0.0610*	
H10	−0.01380	0.14690	0.29320	0.0680*	
H12	−0.12010	−0.00950	0.41260	0.0910*	
H13	−0.21280	0.04330	0.53190	0.0730*	
H14A	−0.00400	0.07850	0.16740	0.1240*	
H14B	0.10790	0.02210	0.16600	0.1240*	
H14C	0.15310	0.07870	0.22920	0.1240*	
HN2	0.571 (2)	0.3049 (8)	0.5344 (15)	0.0490*	
HO2	−0.245 (2)	0.3998 (11)	0.3583 (17)	0.0610*	
H17	0.10200	0.44370	0.45390	0.0500*	
H18	0.35740	0.43970	0.51200	0.0520*	
H20	0.35420	0.25670	0.47390	0.0470*	
H21	0.10130	0.26170	0.41530	0.0520*	
H23	0.60040	0.32340	0.74130	0.0520*	
H24	0.48740	0.34410	0.87520	0.0560*	
H26	0.42660	0.51980	0.79470	0.0620*	
H27	0.53290	0.49820	0.65910	0.0530*	
H28A	0.28250	0.37450	0.96350	0.1500*	
H28B	0.30170	0.42530	1.04000	0.1500*	
H28C	0.44620	0.38870	1.01290	0.1500*	

### Atomic displacement parameters (Å^2^)

**Table:**

	*U*^11^	*U*^22^	*U*^33^	*U*^12^	*U*^13^	*U*^23^
S1	0.0310 (2)	0.0358 (2)	0.0574 (3)	0.0010 (2)	−0.0046 (2)	−0.0053 (2)
O1	0.0374 (6)	0.0350 (6)	0.0519 (8)	0.0030 (5)	−0.0018 (5)	−0.0010 (5)
O2	0.0371 (7)	0.0354 (7)	0.1042 (13)	−0.0012 (6)	−0.0225 (8)	0.0033 (8)
O3	0.0364 (7)	0.0425 (7)	0.0860 (11)	0.0076 (6)	−0.0154 (7)	−0.0127 (7)
O4	0.0517 (8)	0.0546 (9)	0.0638 (9)	−0.0061 (7)	0.0110 (7)	0.0009 (7)
O5	0.1154 (16)	0.0666 (11)	0.0801 (13)	0.0167 (11)	0.0143 (12)	−0.0180 (10)
N1	0.0376 (8)	0.0318 (7)	0.0680 (11)	0.0034 (6)	−0.0133 (7)	−0.0069 (7)
C1	0.0345 (8)	0.0343 (8)	0.0424 (9)	0.0017 (7)	−0.0001 (7)	−0.0041 (7)
C2	0.0330 (8)	0.0352 (8)	0.0358 (8)	0.0011 (6)	−0.0003 (6)	−0.0031 (7)
C3	0.0395 (9)	0.0312 (8)	0.0399 (9)	0.0027 (7)	−0.0009 (7)	−0.0030 (7)
C4	0.0392 (9)	0.0318 (8)	0.0499 (10)	−0.0025 (7)	−0.0032 (7)	−0.0062 (7)
C5	0.0335 (8)	0.0373 (9)	0.0365 (8)	0.0020 (7)	−0.0022 (6)	−0.0059 (7)
C6	0.0423 (9)	0.0312 (8)	0.0500 (10)	0.0009 (7)	−0.0052 (8)	0.0028 (7)
C7	0.0391 (9)	0.0354 (9)	0.0568 (11)	−0.0057 (7)	−0.0061 (8)	0.0014 (8)
C8	0.0355 (8)	0.0331 (8)	0.0481 (10)	0.0000 (7)	−0.0082 (7)	−0.0018 (7)
C9	0.0476 (11)	0.0381 (10)	0.0661 (13)	−0.0008 (8)	−0.0028 (9)	0.0036 (9)
C10	0.0500 (11)	0.0632 (14)	0.0565 (13)	0.0019 (10)	0.0026 (9)	0.0046 (11)
C11	0.0631 (13)	0.0512 (12)	0.0633 (14)	0.0103 (10)	−0.0055 (11)	−0.0105 (11)
C12	0.112 (2)	0.0380 (12)	0.0800 (18)	0.0057 (13)	0.0163 (16)	−0.0088 (11)
C13	0.0842 (16)	0.0351 (10)	0.0653 (14)	0.0013 (10)	0.0117 (12)	0.0018 (10)
C14	0.0737 (17)	0.108 (2)	0.0657 (17)	0.0365 (17)	0.0026 (13)	−0.0108 (16)
S2	0.0325 (2)	0.0355 (2)	0.0537 (3)	−0.0009 (2)	−0.0035 (2)	−0.0059 (2)
O6	0.0353 (7)	0.0463 (8)	0.0725 (10)	0.0065 (6)	−0.0051 (6)	0.0095 (7)
O7	0.0405 (8)	0.0438 (8)	0.1466 (18)	−0.0054 (6)	−0.0317 (9)	0.0155 (10)
O8	0.0321 (6)	0.0509 (8)	0.0756 (10)	0.0035 (6)	−0.0081 (6)	−0.0153 (7)
O9	0.0607 (9)	0.0432 (8)	0.0621 (9)	−0.0048 (7)	0.0111 (7)	0.0019 (7)
O10	0.1012 (14)	0.0737 (11)	0.0574 (10)	0.0305 (10)	0.0240 (9)	0.0128 (9)
N2	0.0356 (8)	0.0331 (8)	0.0602 (10)	0.0064 (6)	−0.0130 (7)	−0.0107 (7)
C15	0.0335 (8)	0.0392 (9)	0.0564 (11)	0.0020 (7)	−0.0007 (8)	0.0085 (8)
C16	0.0310 (8)	0.0389 (9)	0.0403 (9)	0.0020 (7)	0.0004 (7)	0.0061 (7)
C17	0.0392 (9)	0.0365 (9)	0.0489 (10)	0.0081 (7)	−0.0027 (8)	−0.0012 (8)
C18	0.0419 (9)	0.0331 (9)	0.0532 (11)	0.0028 (7)	−0.0077 (8)	−0.0069 (8)
C19	0.0319 (8)	0.0375 (9)	0.0338 (8)	0.0014 (6)	−0.0020 (6)	−0.0020 (7)
C20	0.0347 (8)	0.0300 (8)	0.0527 (10)	0.0043 (6)	−0.0024 (7)	0.0009 (7)
C21	0.0345 (8)	0.0345 (9)	0.0583 (11)	−0.0032 (7)	−0.0043 (8)	0.0023 (8)
C22	0.0338 (8)	0.0303 (8)	0.0464 (10)	−0.0015 (6)	−0.0078 (7)	−0.0021 (7)
C23	0.0412 (9)	0.0286 (8)	0.0571 (11)	0.0022 (7)	−0.0072 (8)	0.0017 (8)
C24	0.0454 (10)	0.0399 (10)	0.0534 (11)	0.0013 (8)	−0.0046 (8)	0.0109 (8)
C25	0.0498 (11)	0.0488 (11)	0.0490 (11)	0.0076 (9)	−0.0020 (9)	0.0021 (9)
C26	0.0661 (13)	0.0323 (9)	0.0548 (12)	0.0104 (9)	0.0021 (10)	−0.0009 (8)
C27	0.0541 (11)	0.0288 (8)	0.0494 (11)	0.0010 (7)	−0.0010 (8)	0.0017 (7)
C28	0.110 (2)	0.119 (3)	0.0770 (19)	0.058 (2)	0.0408 (18)	0.0445 (19)

### Geometric parameters (Å, °)

**Table:**

S1—O3	1.4395 (15)	C3—H3	0.9300
S1—O4	1.4242 (16)	C4—H4	0.9300
S1—N1	1.6249 (17)	C6—H6	0.9300
S1—C8	1.7475 (19)	C7—H7	0.9300
S2—O8	1.4385 (15)	C9—H9	0.9300
S2—O9	1.4245 (16)	C10—H10	0.9300
S2—N2	1.6279 (17)	C12—H12	0.9300
S2—C22	1.7476 (19)	C13—H13	0.9300
O1—C1	1.225 (2)	C14—H14A	0.9600
O2—C1	1.303 (2)	C14—H14B	0.9600
O5—C11	1.375 (3)	C14—H14C	0.9600
O5—C14	1.401 (4)	C15—C16	1.474 (2)
O2—HO1	0.807 (19)	C16—C21	1.390 (2)
O6—C15	1.295 (2)	C16—C17	1.385 (2)
O7—C15	1.223 (2)	C17—C18	1.381 (3)
O10—C25	1.356 (3)	C18—C19	1.393 (2)
O10—C28	1.409 (4)	C19—C20	1.386 (2)
O6—HO2	0.815 (18)	C20—C21	1.368 (3)
N1—C5	1.404 (2)	C22—C27	1.395 (2)
N1—HN1	0.839 (16)	C22—C23	1.378 (3)
N2—C19	1.398 (2)	C23—C24	1.376 (3)
N2—HN2	0.818 (18)	C24—C25	1.391 (3)
C1—C2	1.478 (2)	C25—C26	1.387 (3)
C2—C7	1.397 (3)	C26—C27	1.373 (3)
C2—C3	1.384 (2)	C17—H17	0.9300
C3—C4	1.383 (2)	C18—H18	0.9300
C4—C5	1.392 (2)	C20—H20	0.9300
C5—C6	1.385 (2)	C21—H21	0.9300
C6—C7	1.373 (3)	C23—H23	0.9300
C8—C9	1.382 (3)	C24—H24	0.9300
C8—C13	1.374 (3)	C26—H26	0.9300
C9—C10	1.394 (3)	C27—H27	0.9300
C10—C11	1.370 (3)	C28—H28A	0.9600
C11—C12	1.358 (4)	C28—H28B	0.9600
C12—C13	1.371 (4)	C28—H28C	0.9600
			
O3—S1—O4	119.23 (9)	C11—C10—H10	120.00
O3—S1—N1	103.16 (8)	C11—C12—H12	119.00
O3—S1—C8	109.55 (10)	C13—C12—H12	119.00
O4—S1—N1	110.19 (10)	C12—C13—H13	120.00
O4—S1—C8	107.48 (9)	C8—C13—H13	120.00
N1—S1—C8	106.58 (9)	H14A—C14—H14C	109.00
O9—S2—N2	109.83 (9)	H14B—C14—H14C	109.00
O9—S2—C22	107.95 (9)	O5—C14—H14A	109.00
N2—S2—C22	106.44 (8)	H14A—C14—H14B	109.00
O8—S2—C22	109.55 (9)	O5—C14—H14B	109.00
O8—S2—O9	119.89 (9)	O5—C14—H14C	110.00
O8—S2—N2	102.38 (8)	O6—C15—O7	122.59 (17)
C11—O5—C14	119.0 (2)	O6—C15—C16	117.04 (16)
C1—O2—HO1	108.8 (17)	O7—C15—C16	120.37 (17)
C25—O10—C28	119.1 (2)	C15—C16—C21	117.72 (16)
C15—O6—HO2	108.4 (17)	C15—C16—C17	123.83 (16)
S1—N1—C5	127.10 (13)	C17—C16—C21	118.44 (16)
C5—N1—HN1	116.8 (17)	C16—C17—C18	121.23 (16)
S1—N1—HN1	115.1 (17)	C17—C18—C19	119.35 (16)
S2—N2—C19	128.76 (13)	C18—C19—C20	119.74 (16)
C19—N2—HN2	117.4 (13)	N2—C19—C18	124.27 (16)
S2—N2—HN2	112.5 (13)	N2—C19—C20	115.98 (15)
O1—C1—O2	122.80 (16)	C19—C20—C21	120.06 (16)
O1—C1—C2	123.41 (16)	C16—C21—C20	121.18 (16)
O2—C1—C2	113.78 (15)	S2—C22—C23	118.99 (14)
C3—C2—C7	118.51 (16)	S2—C22—C27	120.76 (14)
C1—C2—C7	120.31 (16)	C23—C22—C27	120.24 (17)
C1—C2—C3	121.11 (16)	C22—C23—C24	120.65 (17)
C2—C3—C4	121.04 (16)	C23—C24—C25	119.21 (18)
C3—C4—C5	119.68 (16)	O10—C25—C26	115.58 (19)
N1—C5—C6	116.62 (15)	O10—C25—C24	124.20 (19)
N1—C5—C4	123.66 (16)	C24—C25—C26	120.22 (19)
C4—C5—C6	119.72 (16)	C25—C26—C27	120.38 (18)
C5—C6—C7	120.13 (17)	C22—C27—C26	119.25 (18)
C2—C7—C6	120.92 (17)	C16—C17—H17	119.00
C9—C8—C13	118.86 (19)	C18—C17—H17	119.00
S1—C8—C13	120.45 (16)	C17—C18—H18	120.00
S1—C8—C9	120.63 (14)	C19—C18—H18	120.00
C8—C9—C10	120.45 (19)	C19—C20—H20	120.00
C9—C10—C11	119.5 (2)	C21—C20—H20	120.00
O5—C11—C10	124.7 (2)	C16—C21—H21	119.00
C10—C11—C12	119.6 (2)	C20—C21—H21	119.00
O5—C11—C12	115.7 (2)	C22—C23—H23	120.00
C11—C12—C13	121.5 (2)	C24—C23—H23	120.00
C8—C13—C12	120.0 (2)	C23—C24—H24	120.00
C4—C3—H3	119.00	C25—C24—H24	120.00
C2—C3—H3	120.00	C25—C26—H26	120.00
C3—C4—H4	120.00	C27—C26—H26	120.00
C5—C4—H4	120.00	C22—C27—H27	120.00
C7—C6—H6	120.00	C26—C27—H27	120.00
C5—C6—H6	120.00	O10—C28—H28A	109.00
C2—C7—H7	120.00	O10—C28—H28B	109.00
C6—C7—H7	120.00	O10—C28—H28C	110.00
C10—C9—H9	120.00	H28A—C28—H28B	110.00
C8—C9—H9	120.00	H28A—C28—H28C	109.00
C9—C10—H10	120.00	H28B—C28—H28C	109.00
			
O3—S1—N1—C5	−175.24 (17)	N1—C5—C6—C7	179.32 (18)
O4—S1—N1—C5	56.43 (19)	C4—C5—C6—C7	−0.5 (3)
C8—S1—N1—C5	−59.89 (19)	C5—C6—C7—C2	0.4 (3)
N1—S1—C8—C9	−56.08 (18)	S1—C8—C9—C10	−178.76 (17)
O3—S1—C8—C9	54.89 (17)	C13—C8—C9—C10	−1.6 (3)
O4—S1—C8—C9	−174.20 (15)	S1—C8—C13—C12	178.0 (2)
N1—S1—C8—C13	126.77 (18)	C9—C8—C13—C12	0.7 (4)
O3—S1—C8—C13	−122.27 (18)	C8—C9—C10—C11	1.2 (3)
O4—S1—C8—C13	8.7 (2)	C9—C10—C11—O5	180.0 (2)
O8—S2—C22—C23	−46.00 (17)	C9—C10—C11—C12	0.0 (4)
O8—S2—C22—C27	134.90 (15)	C10—C11—C12—C13	−0.8 (5)
O9—S2—C22—C23	−178.14 (14)	O5—C11—C12—C13	179.2 (3)
N2—S2—C22—C23	64.00 (16)	C11—C12—C13—C8	0.5 (4)
C22—S2—N2—C19	61.60 (18)	O7—C15—C16—C17	−172.0 (2)
O9—S2—C22—C27	2.76 (17)	O6—C15—C16—C17	8.0 (3)
N2—S2—C22—C27	−115.10 (15)	O6—C15—C16—C21	−170.94 (18)
O9—S2—N2—C19	−55.01 (19)	O7—C15—C16—C21	9.0 (3)
O8—S2—N2—C19	176.56 (16)	C21—C16—C17—C18	−0.1 (3)
C14—O5—C11—C10	4.3 (4)	C17—C16—C21—C20	0.4 (3)
C14—O5—C11—C12	−175.8 (3)	C15—C16—C17—C18	−179.01 (18)
C28—O10—C25—C24	−1.0 (3)	C15—C16—C21—C20	179.36 (18)
C28—O10—C25—C26	178.7 (2)	C16—C17—C18—C19	−0.2 (3)
S1—N1—C5—C4	−13.7 (3)	C17—C18—C19—C20	0.1 (3)
S1—N1—C5—C6	166.56 (15)	C17—C18—C19—N2	178.53 (17)
S2—N2—C19—C20	−169.78 (14)	N2—C19—C20—C21	−178.38 (17)
S2—N2—C19—C18	11.7 (3)	C18—C19—C20—C21	0.2 (3)
O1—C1—C2—C7	−179.84 (17)	C19—C20—C21—C16	−0.4 (3)
O2—C1—C2—C3	−175.85 (17)	S2—C22—C27—C26	178.50 (16)
O2—C1—C2—C7	1.0 (3)	C23—C22—C27—C26	−0.6 (3)
O1—C1—C2—C3	3.4 (3)	S2—C22—C23—C24	−177.51 (14)
C3—C2—C7—C6	−0.1 (3)	C27—C22—C23—C24	1.6 (3)
C1—C2—C3—C4	176.70 (17)	C22—C23—C24—C25	−0.7 (3)
C7—C2—C3—C4	−0.2 (3)	C23—C24—C25—C26	−1.1 (3)
C1—C2—C7—C6	−177.01 (17)	C23—C24—C25—O10	178.56 (18)
C2—C3—C4—C5	0.1 (3)	O10—C25—C26—C27	−177.6 (2)
C3—C4—C5—C6	0.2 (3)	C24—C25—C26—C27	2.2 (3)
C3—C4—C5—N1	−179.58 (17)	C25—C26—C27—C22	−1.3 (3)

### Hydrogen-bond geometry (Å, °)

**Table:**

*D*—H···*A*	*D*—H	H···*A*	*D*···*A*	*D*—H···*A*
N1—HN1···O8^i^	0.84 (2)	2.31 (2)	3.122 (2)	165 (2)
O2—HO1···O7^ii^	0.81 (2)	1.78 (2)	2.583 (2)	172 (2)
N2—HN2···O3^iii^	0.82 (2)	2.15 (2)	2.959 (2)	173.(2)
O6—HO2···O1^iv^	0.82 (2)	2.02 (2)	2.8338 (19)	173 (2)
C4—H4···O4	0.93	2.54	3.149 (2)	123
C7—H7···O2	0.93	2.39	2.709 (2)	100
C9—H9···O7	0.93	2.32	3.224 (3)	165
C13—H13···O4	0.93	2.51	2.883 (3)	105
C13—H13···O10^v^	0.93	2.57	3.381 (3)	146
C14—H14A···O8^iv^	0.96	2.42	3.370 (3)	169
C23—H23···O2	0.93	2.52	3.286 (2)	140
C26—H26···O1^vi^	0.93	2.57	3.197 (2)	125
C27—H27···O9	0.93	2.54	2.905 (3)	104

Symmetry codes: (i) *x*−1, *y*, *z*; (ii) *x*+1, −*y*+1/2, *z*+1/2; (iii) *x*+1, *y*, *z*; (iv) *x*−1, −*y*+1/2, *z*−1/2; (v) −*x*, *y*−1/2, −*z*+3/2; (vi) −*x*+1, *y*+1/2, −*z*+3/2.
